# Potent MERS-CoV Fusion Inhibitory Peptides Identified from HR2 Domain in Spike Protein of Bat Coronavirus HKU4

**DOI:** 10.3390/v11010056

**Published:** 2019-01-14

**Authors:** Shuai Xia, Qiaoshuai Lan, Jing Pu, Cong Wang, Zezhong Liu, Wei Xu, Qian Wang, Huan Liu, Shibo Jiang, Lu Lu

**Affiliations:** 1 Key Laboratory of Medical Molecular Virology of MOE/MOH, School of Basic Medical Sciences and Shanghai Public Health Clinical Center, Fudan University, Shanghai 200032, China; 15111010053@fudan.edu.cn (S.X.); 18111010010@fudan.edu.cn (Q.L.); 17111010015@fudan.edu.cn (J.P.); 16111010068@fudan.edu.cn (C.W.); 17111010065@fudan.edu.cn (Z.L.); xuwei11@fudan.edu.cn (W.X.); wang_qian@fudan.edu.cn (Q.W.); 2 State Key Laboratory of Virology, Wuhan Institute of Virology, Chinese Academy of Sciences, Wuhan 430071, China; 3 Lindsley F. Kimball Research Institute, New York Blood Center, New York, NY 10065, USA

**Keywords:** MERS-CoV, fusion inhibitor, peptide, cell–cell fusion, HKU4

## Abstract

The Middle East respiratory syndrome coronavirus (MERS-CoV) emerged in 2012 and caused continual outbreaks worldwide with high mortality. However, no effective anti-MERS-CoV drug is currently available. Recently, numerous evolutionary studies have suggested that MERS-CoV originated from bat coronavirus (BatCoV). We herein reported that three peptides derived from the HR2 region in spike protein of BatCoV HKU4, including HKU4-HR2P1, HKU4-HR2P2 and HKU4-HR2P3, could bind the MERS-CoV HR1-derived peptide to form a six-helix bundle (6-HB) with high stability. Moreover, these peptides, particularly HKU4-HR2P2 and HKU4-HR2P3, exhibited potent inhibitory activity against MERS-CoV S-mediated cell–cell fusion and viral infection, suggesting that these HKU4 HR2-derived peptides could be candidates for futher development as antiviral agents against MERS-CoV infection.

## 1. Introduction

**Figure 1:**
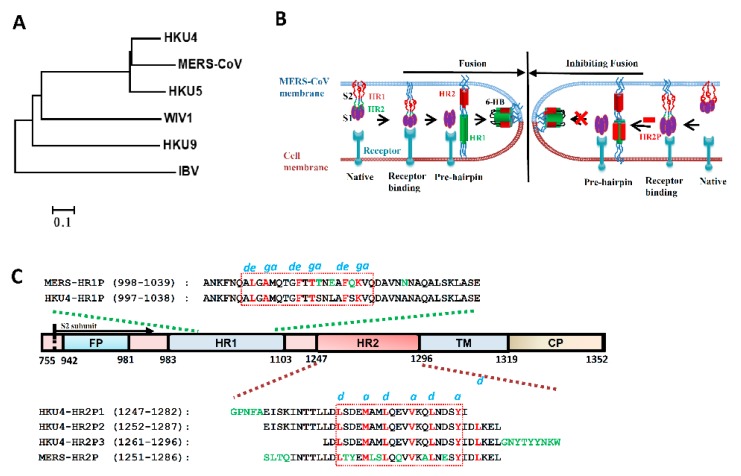
The design of HKU4 HR2-derived peptides. (**A**) Neighbor-joining tree created with the spike sequence of representative bat-CoVs and Infectious Bronchitis Virus (IBV). (**B**) The antiviral mechanism of HR2-derived peptides. (**C**) Schematic representation of HKU4 S protein S2 subunit. FP, fusion peptide; HR, heptad repeat domain; TM, transmembrane domain; CP, cytoplasmic domain. Corresponding sequences of the designed peptides (HKU4-HR1P and HKU4-HR2Ps) are shown in the diagram. The helical region in the HR1- and HR2-derived peptides is highlighted in the red boxes, according to the sequences of MERS-HR1P and MERS-HR2P. The identical amino acids at the *e* and *g* positions in HR1 and those at *a* and *d* positions in HR2 of MERS-CoV and HKU4 are highlighted in red. The different amino acids in HR1Ps and HR2Ps between MERS-CoV and HKU4 are highlighted in green.

Middle East respiratory syndrome coronavirus (MERS-CoV), a novel human coronavirus (Figure 1A), emerged in Saudi Arabia in 2012 [1], rapidly spread wordwide, and caused continuous outbreaks with significant mortality and morbidity [2,3]. As of 27 November 2018, the World Health Organization (WHO) reported 2266 laboratory-confirmed cases of infection with MERS-CoV and 804 MERS-associated deaths (about 36% fatality rate) in 27 countries (available online: http://www.who.int/emergencies/mers-cov/en/). MERS-CoV uses dipeptidyl peptidase-4 (DPP4, also named CD26), a type-II transmembrane glycoprotein, as the cellular receptor to infect humans and cause severe respiratory disease and other severe complications, including renal failure and even multiorgan failure [4,5,6,7,8]. However, no licensed vaccines or effective therapeutics against MERS-CoV infection have been approved by the U.S. Food and Drug Administration (FDA). This calls for the urgent development of effective therapeutics against MERS-CoV infection for clinical use.

Similar to other coronaviruses [9], MERS-CoV is an enveloped positive-sense single-stranded RNA virus and belongs to the lineage C betacoronavirus in the family coronaviridae [10]. MERS-CoV displays its spike protein (S), a type I transmembrane glycoprotein, on its enveloped membrane surface in a trimer state. S protein contains two subunits: S1 subunit (S1) and S2 subunit (S2). S1 recognizes and binds the cellular receptor, while S2 mediates viral and cellular membrane fusion. The interaction between the receptor-binding domain (RBD) in S1 and the receptor (DPP4) on the host cell surface induces S2 structural changes, including the insertion of N-terminal fusion peptide into the host cell membrane, the formation of a homotrimer by heptad repeat 1 (HR1) helices, exposing three hydrophobic grooves on the surface, and the binding of three heptad repeat 2 (HR2) molecules to the hydrophobic grooves of HR1 trimer to form a six-helix bundle (6-HB), which brings the viral and cell membranes close together for fusion. Finally, MERS-CoV releases its genetic materials into target cells (Figure 1B) [11,12]. Disrupting the process of MERS-6HB formation can inhibit virus–cell membrane fusion and abolish viral infection [13,14]. Our previous studies found that a peptide, MERS-HR2P, derived from the MERS-CoV HR2 region, could prevent the formation of the fusion core by competitively binding to HR1 and blocking the native interaction between HR1 and HR2 (Figure 1B). MERS-HR2P has no effect on SARS-CoV infection, and SARS-CoV HR2-derived peptide, CP-1, failed to inhibit MERS-CoV infection [14,15], suggesting that MERS-HR2P is specific for inibiting MERS-CoV infection. Whether other novel and potent peptide fusion inhibitors can be developed against MERS-CoV from other coronavirus HR2 regions is still unclear. 

Although camels are the intermediate host for the transmission of MERS-CoV, bats are likely to be its original source and serve as the natural host [10,16,17,18]. Phylogenetically, MERS-CoV is very closely related to *Tylonycteris sp. Bat CoV* HKU4 (Ty-BatCoV HKU4) (Figure 1A). Similar to MERS-CoV, HKU4 also harbors the ability to use human DPP4 as the receptor for infecting the target cell [19,20]. We herein identified that HKU4-HR2P1, HKU4-HR2P2 and HKU4-HR2P3 peptides, derived from the HKU4 HR2 region, could bind MERS-HR1P, a MERS-CoV HR1-derived peptide, and mimic the interaction of MERS-CoV HR1 and HR2 regions to form stable 6-HB. Moreover, these peptides exhibited significant inhibitory activity in both MERS-CoV S-mediated cell–cell fusion assay and pseudovirus infection assay, with even more potency than that of the reported peptide, MERS-HR2P. Taken together, these HKU4 HR2-derived peptides could be considered as candidates for the development of anti-MERS-CoV agents for clinical use. 

## 2. Materials and Methods

### 2.1. Cells, Viruses and Peptides

Huh-7 and 293T cells were obtained from the Chinese Academy of Sciences Cell Bank (Shanghai, China). All cell lines were cultured in Dulbecco’s Modified Eagle’s Medium (DMEM) with 10% fetal bovine serum (FBS). Plasmids, including pcDNA3.1-MERS-S, pcDNA3.1-MERS-S with Q1020H or Q1020R mutation, pAAV-IRES-S-GFP and pNL4-3.Luc.R-E, were constructed in our laboratory. All peptides were synthesized by KareBay Biochem (Monmouth Junction, NJ, USA) with HPLC purity >90% [21,22]. The average hydrophilicity of peptides was predicted by using an online peptide-calculator program (available online: http://www.bachem.com/service-support/peptide-calculator/).

### 2.2. Coronavirus Phylogenetic Analysis

Phylogenetic tree was constructed using MEGA6.06. Accession numbers utilized for phylogenetic analysis are as follows: BtCoV-HKU4 (NC_009019.1), BtCoV-HKU5 (NC_009020), MERS-CoV (AID55097.1), BtSCoV-WIV1 (KF367457), BtCoV-HKU9 (EF065516), and IBV (KY421672).

### 2.3. Circular Dichroism Spectroscopic Analysis

Circular dichroism spectra (198-260 nm) were collected on a J-815 spectropolarimeter (Jasco, Inc., Tokyo, Japan) to evaluate the secondary structure of the individual peptide or the complexes dissolved in phosphate-buffered saline (PBS) with the final concentration at 10 μM [15,23]. The ellipticity value of −33,000 deg cm^2^ dmol^−1^ at 222 nm was taken as 100% α-helicity [24,25]. Thermal denaturation of peptide complexes was monitored from 20 °C to 100 °C at 222 nm with a thermal gradient of 5°C/ min. The midpoint of the thermal unfolding transition (Tm) values was acquired by Jasco software utilities.

### 2.4. Native Polyacrylamide Gel Electrophoresis (N-PAGE)

Native polyacrylamide gel electrophoresis (N-PAGE) was conducted as described elsewhere [15]. Briefly, each HKU4 HR2-derived peptide (40 µM) in PBS was incubated with HKU4-HR1P or MERS-HR1Ps (20, 40, 80 µM), respectively, at 37°C for 30 min, using PBS as control, and then loaded on a tris-glycine gel (12%) with tricine glycine running buffer (pH 8.6). Finally, staining was performed with Coomassie blue, and the images were visualized on the FluorChem Imaging System (Alpha Innotech/ProteinSimple).

### 2.5. Cell–Cell Fusion Assay

The assay for MERS-CoV S protein-mediated cell–cell fusion was performed as described elsewhere [14,26,27,28]. Briefly, 293T cells transiently co-expressing MERS-CoV S protein and GFP protein on cell surface and in cytoplasm, respectively, were used as the effector cells (293T/S/GFP), and Huh-7 cells were used as the target cells. Then the effector cells (1 × 10^4^ cells per well) and target cells (5 × 10^4^ cells per well) were cocultured in the wells of a flat-bottom 96-well plate at 37 °C for 2 h in the presence or absence of peptides at the indicated concentrations. Finally, the fused and unfused cells were visualized, photographed and counted under an inverted fluorescence microscope (Nikon Eclipse Ti-S). PBS without peptides was used as no inhibition control, and the median inhibitory concentration (IC_50_) was calculated using the CalcuSyn software [29].

### 2.6. Generation and Packaging of Middle East Respiratory Syndrome Coronavirus (MERS-CoV) Pseudovirus

The package of MERS-CoV pseudovirus was described in our previous studies [14,27]. Briefly, we cotransfected 293T cells with plasmid pcDNA3.1-MERS-S with or without Q1020H/R mutation and plasmid pNL4-3.luc.R-E encoding Env-defective, luciferase-expressing HIV-1 [30]. Then, the pseudoviruses in the supernatant were collected 48–72 h post-transfection and quantified by the level of lentivirus p24. Target cells, Huh-7, were preplated in 96-well plates (1 × 10^4^ cells per well). The MERS-CoV pseudovirus was premixed with peptide at indicated concentration and incubated for 30 min at 37 °C. PBS was used as no inhibition control. Then, the mixture was added to the Huh-7 cells, replaced by fresh medium 12 h post-infection, and then incubated for an additional 48 h. Transduced Huh-7 cells were lysed for detection of relative light units (RLU) according to the luciferase assay system manual (Promega, Madison, WI, USA) [31]. 

### 2.7. Time-of-Addition Assay

The time-of-addition assay was conducted as described in our previous study [14]. Briefly, Huh-7 cells plated in 96-well plates were incubated with MERS-CoV PsV, while each peptide at a final concentration of 10 μM was added 0, 0.5, 1, 2, 4 or 6 h post-infection. Cells were lysed 72 h later to determine the entry inhibition ratio.

### 2.8. Time-of-Removal Assay

Each peptide (10 µM) was added to Huh-7 cells, followed by an incubation at 37 °C for 30 min, respectively. The cells were then washed with PBS to remove the unbound peptide and the cells that were not washed were used as control, followed by addition of the MERS-CoV pseudovirus. After co-culture at 37 °C for 3 days, cells were lysed to test the inhibitory activity of peptides.

### 2.9. Cytotoxicity Assay

The cytotoxicity of peptides to Huh-7 cells was measured following the instructions in the manual provided in the Cell Counting Kit-8 (CCK-8; Dojindo, Kumamoto, Kyushu, Japan) [22]. Briefly, each peptide with indicated concentrations was mixed with 10^4^ target cells in each well of 96-well plates to co-incubate for 48 h. Then culture medium was replaced by fresh cell medium with 4% CCK-8 solution for an additional incubation 2 h at 37 °C. Finally, the absorbance was detected at 450 nm (A450).

## 3. Results

### 3.1. Design of HKU4 HR2-Peptides 

Our previous studies crystallized the fusion core structure of the MERS-CoV S2 subunit and designed peptides MERS-HR1P and MERS-HR2P based on the fusion structure [14]. These peptides can mimic the interaction of HR1 and HR2 regions to form 6-HB [14]. Through multiple sequence alignment with the MERS-CoV S protein S2 subunit, we found that the S2 subunit of HKU4 also harbors a fusion peptide (FP, residues 942–981), an HR1 domain (residues 983–1103), an HR2 domain (residues 1247–1296), a transmembrane domain (residues 1296–1319) and an intracellular domain (residues1319–1352). The designated HKU4-HR1P spans residues 997–1038 in the HR1, while peptides HKU4-HR2P1, HKU4-HR2P2, HKU4-HR2P3 span residues 1247–1282, 1252–1287, and 1261–1296, respectively (Figure 1C). Similar to MERS-HR2P, the three HKU4 HR2-derived peptides all contain the helical region (residues1263–1281), which contains some different amino acids, such as 1264S, 1265D, 1268A, 1269M, 1272E, 1276Q and1279D (Figure 1C). However, the *a* and *d* residues in the HKU4 HR2 helical region, which are fully the same as those of the MERS-CoV HR2 helical region, are responsible for interaction with the *g* and *e* residues in the HR1 region, correspondingly [14], suggesting that the HKU4 HR2-derived peptides might cross-target the MERS-CoV HR1 region, as does MERS-HR2P. 

### 3.2. Interaction between MERS-HR1P and HKU4 HR2-Derived Peptides

**Figure 2:**
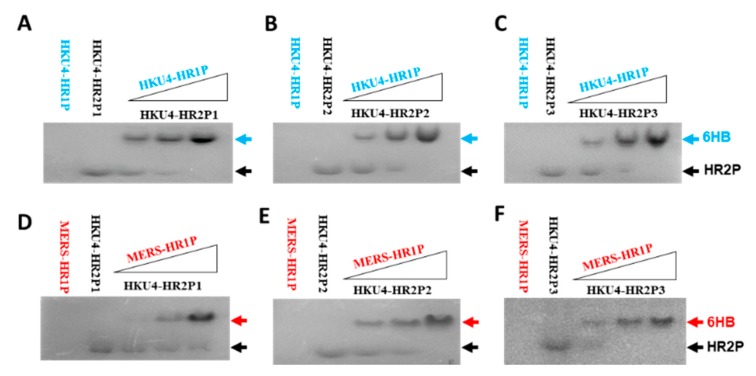
Determination of the interactions between HKU4-HR1P with HKU4-HR2P1 (**A**), HKU4-HR2P2 (**B**) and HKU4-HR2P3 (**C**), as well as the interaction between MERS-HR1P with HKU4-HR2P1 (**D**), HKU4-HR2P2 (**E**) and HKU4-HR2P3 (**F**) by native polyacrylamide gel electrophoresis (N-PAGE).

To investigate whether the HKU4-HR2Ps could interact with HKU4-HR1P to mimic the HR1 and HR2 regions, we determined their interaction by using N-PAGE. After coincubation of HKU4-HR1P at 20, 40, and 80 µM and HKU-HR2P1 (or HKU1-HR2P2 or HKU4-HR2P3) in PBS at 40 µM, respectively, the samples were loaded into the tris-glycine gel for electrophoresis. As shown in Figure 2A–C, individual HKU4 HR2-derived peptides moved down to the lowest part of the gel under native electrophoresis, mainly by their carried negative charges. On the contrary, HKU4-HR1P with positive charges could not move into the gel. Meanwhile, the mixtures of HKU4-HR1P and each HKU4 HR2-derived peptide showed new bands at the upper part in the gel (lanes 3, 4 and 5), confirming that all three HKU4-HR2 peptides could, indeed, bind to HKU4-HR1P.

We subsequently assessed the ability of HKU4 HR2-derived peptides to bind the MERS-CoV HR1 region, thereby allowing us to detect the interaction between HKU4-HR2Ps and MERS-HR1P, which was derived from the MERS-CoV HR1 region. We saw that MERS-HR1P did interact with all three HKU4-HR2 peptides and form new bands on N-PAGE, as well as HKU4-HR1P (Figure 2D,E), suggesting that the HKU4-HR2 peptides could cross-target the MERS-CoV HR1 region. 

**Figure 3:**
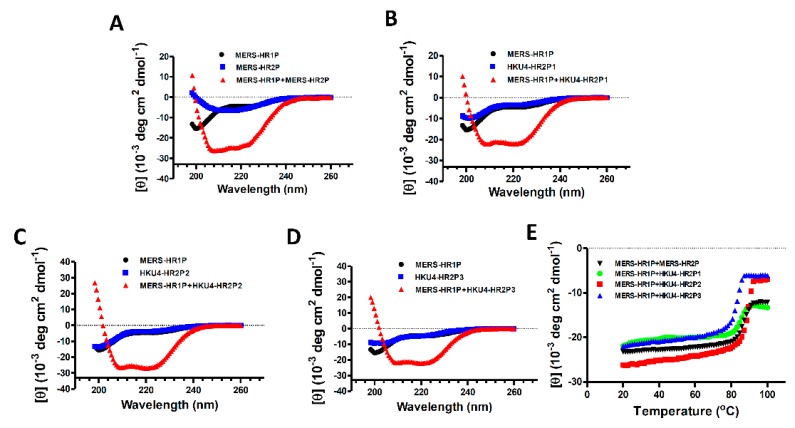
Circular dichroism (CD) spectra of MERS-HR1P, MERS-HR2P and MERS-HR1P/MERS-HR2P complex (**A**), MERS-HR1P, HKU4-HR2P1 and MERS-HR1P/ HKU4-HR2P1 complex (**B**), MERS-HR1P, HKU4-HR2P2 and MERS-HR1P/ HKU4-HR2P2 complex (**C**), MERS-HR1P, HKU4-HR2P3 and MERS-HR1P/ HKU4-HR2P3 complex (**D**) in phosphate buffer (pH 7.2). (**E**) Melting curves of the complexes formed by MERS-HR1P and MERS-HR2P, HKU4-HR2P1, HKU4-HR2P2, or HKU4-HR2P3, respectively.

To characterize the interaction between MERS-HR1P and those HR2-derived peptides, we used circular dichroism (CD) to determine the secondary structures of each peptide and their complexes. As shown in Figure 3A–D, each peptide (MERS-HR2P, HKU4-HR2P1, HKU4-HR2P2, HKU4-HR2P3 or MERS-HR1P) exhibited a low α-helicity structure (10.5%-13.5%), whereas the CD spectrum of HKU4-HR2P1/MERS-HR1P, HKU4-HR2P1/MERS-HR1P, HKU4-HR2P2/MERS-HR1P and HKU4-HR2P3/MERS-HR1P complexes display the characteristic α-helical structure, i.e., double negative peak at 208 and 222 nm, and possess helicity at 70.9%, 66.4%, 80.3% and 66.8%, respectively. Consistent with our previous studies [14], the complex of MERS-HR1P/MERS-HR2P showed high thermostability with the Tm value of 87.1 °C. Similarly, the complexes formed by MERS-HR1P and HKU4-HR2P peptides also possess strong thermostability with Tm values ranging from 83.5 to 90.5 °C (Figure 3E). These results show that all three HKU4-HR2 peptides can bind MERS-HR1P and form 6-HB formation with good stability. Among them, HKU4-HR2P2 exhibited the highest binding affinity to MERS-HR1P and formed the most α-helicity, suggesting that it alone might have the most potent anti-MERS-CoV effect. 

### 3.3. Inhibition of MERS-CoV S Protein-Mediated Cell–Cell Fusion

**Figure 4:**
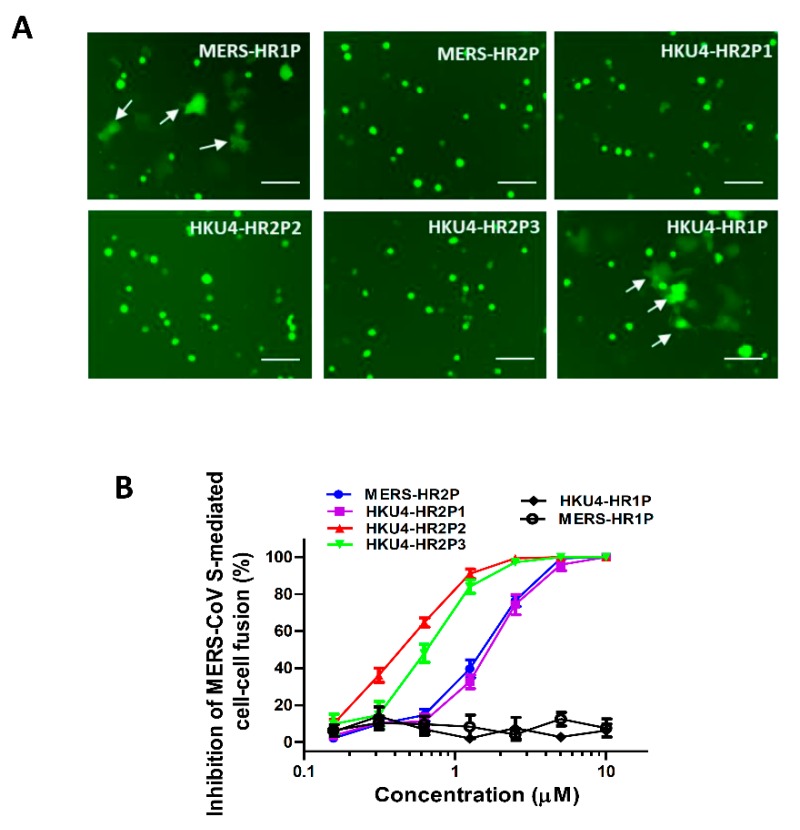
Inhibitory activity of peptides as demonstrated by MERS-CoV S-mediated cell–cell fusion assay. (**A**) Images of MERS-CoV S protein-mediated cell–cell fusion in the presence of MERS-HR1P, MERS-HR2P, HKU4-HR2P1, HKU4-HR2P2, HKU4-HR2P3 and HKU4-HR1P, respectively, all at the concentration of 10 μM. Arrows show the fused cells. Scale bars, 800 μm. (**B**) Inhibitory activities of MERS-HR2P, HKU4-HR2P1, HKU4-HR2P2, HKU4-HR2P3, HKU4-HR1P and MERS-HR1P against MERS-CoV S-mediated cell–cell fusion. Each sample was tested in triplicate and the data are expressed as means ± standard deviation (SD). Each experiment was repeated twice and similar results were obtained.

To determine whether the peptides could inhibit MERS-CoV S-mediated cell–cell fusion, we tested them with a MERS-CoV S-mediated cell–cell fusion assay, in which the GFP of effector cells diffused into the target cells such that the fused cells showed larger size and weaker fluorescence than unfused effector cells. Similar to MERS-HR2P, HKU4-HR2P1, HKU4-HR2P2 and HKU4-HR2P3 peptides could effectively inhibit MERS-CoV S-mediated cell–cell fusion with IC_50_s at concentrations of 1.09, 0.38 and 0.55 μM, respectively (Figure 4A,B, and Table 1). However, HKU4-HR1P did not show fusion-inhibitory activity comparable to that of MERS-HR1P, whereas HKU4-HR2P2 and HKU4-HR2P3 showed more potent inhibitory activity than that of MERS-HR2P (IC50 = 1.07 μM), which was, in fact, derived from the MERS-CoV HR2 region, indicating that HKU4-HR2P2 and HKU4-HR2P3 are good candidates for development as potent anti-MERS-CoV agents.

### 3.4. Inhibition of Pseudotyped MERS-CoVs Infection

**Figure 5:**
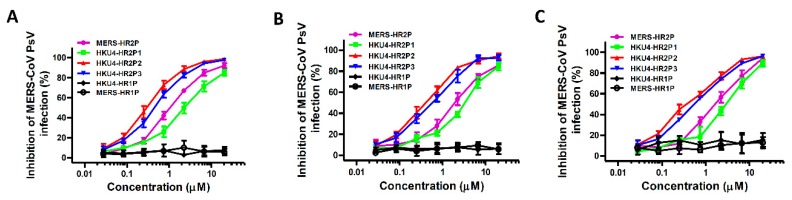
Inhibitory activity of peptides as demonstrated by pseudotyped MERS-CoV infection mediated by S protein (**A**) or S protein with Q1020H (**B**) or Q1020R (**C**). Each sample was tested in triplicate and the data are expressed as means ± SD. Each experiment was repeated twice and similar results were obtained.

To further assess the anti-MERS-CoV activities of the HKU4 HR2-derived peptides, we tested them using a MERS-CoV pseudovirus infection assay, which is able to mimic the entry process of authentic MERS-CoV and has been widely used in basic and antiviral research [14]. HKU4-HR2P1, HKU4-HR2P2 and HKU4-HR2P3 peptides inhibited entry of MERS-CoV in a dose-dependent manner with IC_50_s of 2.15, 0.34 and 0.48 μM (Figure 5A and Table 1), respectively, and did so in a manner comparable with that of MERS-HR2P (IC_50_ = 1.14 μM), whereas neither MERS-HR1P nor HKU4-HR1P exhibited inhibitory activity. The results from the time-of-addition assay indicated that the inhibitory activity of HKU4-HR2P2 and HKU4-HR2P3 on pseudotyped MERS-CoV infection was significantly decreased when the peptides were added to cells 4 h post-infection (Figure S1), suggesting that these peptides target the viral fusion and entry stages.

Based on numerous epidemiological studies, researchers have found some mutant sites in the spike protein of MERS-CoV, in particular Q1020, which is under strong positive selection in the S2 subunit [32,33]. More importantly, Q1020 is located at the MERS-CoV HR1 region, which is the target site for peptide fusion inhibitors, including HKU4 HR2-derived peptides, and might affect the antiviral activity of these peptides. Therefore, to further assess whether these new peptides would possess broad-spectrum anti-MERS-CoV activity, we used the pseudotyped S-mediated MERS-CoVs with Q1020H and Q1020R mutant sites to infect target cells in the presence of each peptide. As shown in Figure 5B,C, similar to MERS-HR2P, the three HKU4-HR2 peptides could effectively inhibit infection caused by the two pseudotyped MERS-CoV mutants. Again, HKU4-HR2P2 and HKU4-HR2P3 exhibited more potent inhibitory activity than either that of MERS-HR2P or HKU4-HR2P1. However, these peptides exhibited no obvious inhibition on vesicular stomatitis virus (VSV) G protein-mediated pseudovirus infection at the concentration up to 10 μM (Figure S2A). The results from a time-of-removal assay indicated that the inhibitory activity of the peptides on MERS-CoV pseudovirus is not associated with the binding of the peptides to the target cells (Figure S2B). Besides, these peptide at high concentration (100 μM) showed no cytotoxicity (Figure S2C). All these results suggest that the fusion inhibitory activity of these peptides is not because of their non-specific interaction with the viral or cellular membranes or proteins, nor their cytotoxicity to the target cells.

## 4. Discussion

In the early 1990s, Jiang et al and Wild et al identified potent HIV-1 fusion inhibitory peptides derived from the HIV-1 gp41 CHR (or HR2) domain, SJ-2176 and DP-178 (it was later renamed as T-20) [34,35]. Subsequently, there have been numerous reports showing that peptides derived from the HR2 domain in the class I membrane fusion proteins of some enveloped viruses, such as respiratory syncytial virus (RSV) [36], paramyxoviruses simian virus 5 (SV5) [37], Nipah virus [38], and mouse hepatitis virus (MHV) [39], exhibit inhibitory activity against the corresponding viruses. Similarly, in our previous work, we found that both MERS-HR2P, derived from MERS-CoV HR2 region, and CP-1, derived from SARS-CoV HR2 region, exhibited effective fusion-inhibitory activity against MERS-CoV and SARS-CoV, respectively [14,15]. However, MERS-HR2P had no inhibitory activity against SARS-CoV, and SARS-HR2P (CP-1) had no inhibitory activity on MERS-CoV, suggesting that the HR2-derived peptides lacked cross-inhibitory activity, thus limiting the development of broad HCoV fusion inhibitors. To the best of our knowledge, we herein report, for the first time, that BatCoV HR2-derived peptides, namely HKU4-HR2P1, HKU4-HR2P2 and HKU4-HR2P3, could successfully cross-bind MERS-HR1P to form stable 6-HB. These peptides, especially HKU4-HR2P2 and HKU4-HR2P3, exhibited potent inhibitory activity against MERS-CoV S-mediated cell–cell fusion and effectively blocked pseudotyped MERS-CoV infection, indicating the existence of a potent fusion inhibitor against MERS-CoV infection in the natural BatCoV HR2 region. 

We previously reported that the fusion core region of HR1 and HR2 is important for the formation of 6-HB [14]; therefore, we herein designed HKU4 HR2-derived peptides containing the helical region. All peptides exhibited antiviral activity against MERS-CoV infection. Apart from HR2 helices, previous studies also suggested that the N- and C-terminal tail regions of HR2 could be important to the inhibitory activity of peptides [40]. Indeed, the three HKU4 HR2-derived peptides possess different terminal tail regions and exhibited various antiviral activity against MERS-CoV infection. On the other hand, the location of HKU4-HR2P2 in the HKU4 HR2 region is the same as that of MERS-HR2P in the MERS-CoV HR2 region. However, the higher helicity (80.3%) and Tm value (90.5 °C) of the HKU4-HR2P2/MERS-HR2 complex than those of the MERS-HR2P2/MERS-HR2 complex may contribute to the higher anti-MERS-CoV activity of HKU4-HR2P2 than that of MERS-HR2P. 

More interestingly, the values of helicity and Tm of the HKU4-HR2P3/MERS-HR1P complex are equal to those of HKU4-HR2P1/MERS-HR1P, but lower than those of MERS-HR2P/MERS-HR1P, while HKU4-HR2P3 showed more potent antiviral activity compared to either HKU4-HR2P1 or MERS-HR2P. The precise antiviral mechanism is worth elucidating in future studies, but it can be speculated here that the answer might lie in the C-terminal tail of HKU4-HR2P3, which belongs to the tryptophan (Trp)-rich membrane proximal external region (MPER). This is best exemplified by SARS-CoV MPER-derived peptide, which can interact with the internal fusion peptide (IFP23) to inhibit the formation of MPER-IFP heteromer, a putative quaternary structure that extends from the 6-HB and functions in membrane fusion [41,42]. Moreover, the MPER-derived peptide could directly inhibit the SARS-CoV entry. HKU4-HR2P3 exhibited similar potent inhibitory activity likely from the blocking of MERS-CoV 6-HB formation and disturbance of the interaction between MPER and IFP in MERS-CoV infection. This clearly constitutes a novel strategy for the development of MERS-CoV peptide fusion inhibitors with bispecific targets: HR1 and IFP.

Consistent with the reports that unlike the HR2 (or CHR) peptides, the HR1 (or NHR) peptides derived from the enveloped viruses with class I membrane fusion proteins (e.g., HIV-1, SARS-CoV, and MERS-CoV) have no or low activity to inhibit 6-HB formation, cell–cell fusion, and viral infection, mainly because these HR1 peptides tend to aggregate in physiological solutions [14,15,43,44,45], we found that MERS-HR1P and HKU4-HR1P also exhibited no fusion inhibitory activity, possibly for the same reason described above. Nonetheless, we have previously reported that conjugation of the T4 fibritin trimerization domain, Foldon (Fd), to the HR1 (or NHR) peptides derived from the HIV-1 gp41, such as N36Fd and N28Fd, make the NHR peptides fold into stable and soluble trimeric coiled-coils with potent HIV-1 fusion inhibitory activity [46]. We will use a similar approach to construct MERS-HR1P-Fd and HKU4-HR1P-Fd with MERS-CoV and HKU4 fusion inhibitory activity, respectively. 

The HKU4 coronavirus is closely related to MERS-CoV, and it can recognize and bind the same receptor, human DPP4 [47]. More importantly, the risk of infecting humans could be sharply increased through the simple induction of two mutants in S protein [48]. However, only sparse information about the HKU4 fusion core is currently available, and no effective fusion inhibitors have been reported. Here, based on the structure of the MERS-CoV 6-HB fusion core, we designed HKU4-HR1P and HKU4-HR2P peptides, which can interact to mimic the formation process of 6-HB by their own HR1 and HR2 regions. Hence, this study contributes to a better understanding about the mechanism of HKU4 infection and provides more information for the development of HKU4 peptide fusion inhibitors in the event that HKU4 crosses the species barrier to infect humans. 

In summary, we have developed a series of bat-CoV-derived peptide fusion inhibitors, including HKU4-HR2P1, HKU4-HR2P2 and HKU4-HR2P3, which can interact with MERS- HR1P to form highly stable 6-HB, mimicking the formation of MERS-CoV 6-HB structure. Additionally, these peptides, especially HKU4-HR2P2 and HKU4-HR2P3, exhibited potent inhibitory activity against MERS-CoV infection, indicating that these bat-CoV peptides are promising as candidates for development as clinical agents against MERS-CoV infection. 

## Figures and Tables

**Table 1:** Solubility and inhibitory activities of peptides.

Peptide	Average Hydro-Philicity	Solubility (μM) in				Inhibitory Activity of Peptides, IC_50_ (μM)							
		PBS (pH 7.2)		Water		Cell–Cell Fusion		Pseudovirus Infection					
								Q1020		Q1020H		Q1020R	
		Mean	SD	Mean	SD	Mean	SD	Mean	SD	Mean	SD	Mean	SD
HKU4-HR2P1	0.0	1569	110	651	40	1.09	0.21	2.15	0.17	2.72	0.59	2.42	0.93
HKU4-HR2P2	0.2	2924	131	150	25	0.38	0.01	0.34	0.06	0.44	0.1	0.3	0.04
HKU4-HR2P3	0.0	118	13	1674	73	0.55	0.06	0.48	0.08	0.52	0.1	0.4	0.03
MERS-HR2P	−0.2	105	12	29	14	1.07	0.22	1.14	0.02	1.71	0.02	1.31	0.07

IC_50_: Concentration of peptide at which 50% of HCoV S-mediated cell–cell fusion was blocked.

## Supplementary Materials

Supplementary materials can be found at http://www.mdpi.com/1999-4915/11/1/56/s1.
