# Supplementary material for: Potent MERS-CoV Fusion Inhibitory Peptides Identified from HR2 Domain in Spike Protein of Bat Coronavirus HKU4
Source: Viruses. 2019 Jan 14;11(1):56. doi: 10.3390/v11010056 (PMC6357153; doi:10.3390/v11010056)
Supplement: Supplementary file 1 [file viruses-11-00056-s001.pdf]

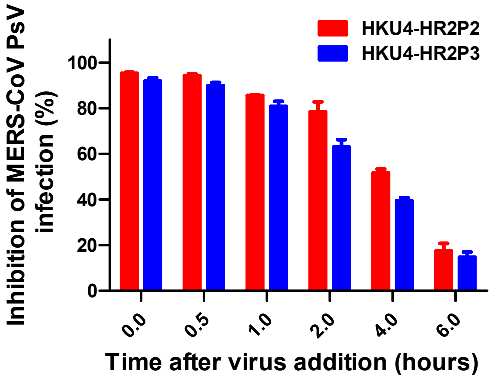

Figure S1. Inhibition of HKU4-HR2P2 and HKU4-HR2P3 on pseudotyped MERS-CoV infection at various intervals post-infection.

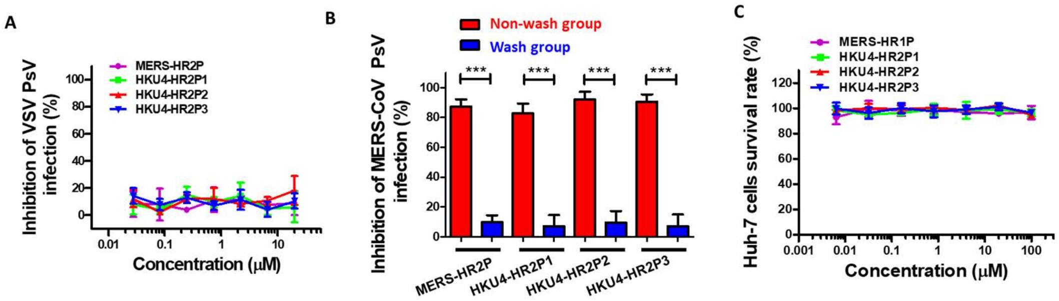

Figure S2. The antiviral specificity of the HKU4-HR2 peptides. (A) VSV-G pseudovirus infection assay. HKU4-HR2 peptides had no inhibitory activity against VSV-G pseudovirus infection as well as MERS-HR2P. (B) Time-of-removal assay. The antiviral activity of HKU4-HR2 peptides is not because of their interaction with the target cells (Huh-7). *** represents $p < 0.001$. (C) Cytotoxicity assay. The antiviral activity of HKU4-HR2 peptides is not because of their cytotoxicity to the target cells (Huh-7).
